# Chemical Composition and Functional Effects of Oilseed Cakes on Redox Balance, Immune Function, Growth Performance, and Carcass Characteristics in Swine Production: A Review

**DOI:** 10.3390/ani16142266

**Published:** 2026-07-22

**Authors:** Nikola Pietrzak, Anita Zaworska-Zakrzewska, Piotr Janiszewski, Iwona Sembratowicz, Anna Czech

**Affiliations:** 1 Department of Meat and Fat Technology, Prof. Wacław Dąbrowski Institute of Agricultural and Food Biotechnology, 60-111 Poznan, Poland; pietrzak.n.k@gmail.com (N.P.); piotr.janiszewski@ibprs.pl (P.J.); 2 Department of Animal Nutrition, Faculty of Veterinary Medicine and Animal Science, Poznan University of Life Sciences, Wołyńska 33, 60-637 Poznan, Poland; 3 Department of Biochemistry and Toxicology, University of Life Sciences in Lublin, Akademicka 13, 20-950 Lublin, Poland; iwona.sembratowicz@up.edu.pl

**Keywords:** oilseed cake, functional feed ingredients, redox balance, oxidative stress, immune modulation, local alternative feed, pig performance

## Abstract

Oilseed cakes (OC), generated as by-products of mechanical oil extraction, are increasingly recognized as functional feed ingredients in swine production. Besides providing protein and residual lipids, they contain bioactive compounds such as polyphenols, tocopherols, and phytosterols that may exert antioxidant and immunomodulatory effects. This narrative review is based on a narrative literature search conducted in the Web of Science, Scopus, PubMed, and Google Scholar databases, covering publications from 2000 to 2026. This review summarizes current knowledge on the role of OC in pig nutrition, focusing on redox balance, immune modulation, and production performance. Experimental studies indicate that rapeseed, soybean, sunflower, and other oilseed cakes can influence oxidative stress markers and antioxidant enzyme activity, contributing to protection against oxidative damage. Evidence also suggests that these by-products may affect immune responses through improvements in intestinal barrier integrity and modulation of cytokine production. Among the reviewed OC, rapeseed, soybean, sunflower, and flaxseed cakes showed the most consistent evidence of beneficial effects on antioxidant status, immune function, and production performance, particularly when included at moderate dietary levels. In contrast, the available evidence for camelina, cottonseed, Amarula, and high-fiber oilseed by-products remains limited or inconsistent. Their effects appear to depend largely on dietary inclusion level, processing method, and the presence of antinutritional factors. Excessive dietary inclusion may impair nutrient digestibility and growth performance, highlighting the importance of optimizing inclusion levels according to the specific type of OC. Overall, OC represent a promising component of sustainable pig-feeding strategies, supporting animal health and production efficiency while promoting the use of agro-industrial by-products.

## Simple Summary

Oilseed cakes (OC) are by-products of vegetable oil production and represent valuable sources of protein, energy, and bioactive compounds for animal feeding. In swine nutrition, they are increasingly considered sustainable alternative feed ingredients. Besides their nutritional value, OC contain compounds such as polyphenols and tocopherols that may influence antioxidant status, immune responses, and pig health. However, these effects have been investigated separately rather than in an integrated manner. This review summarizes current knowledge on the functional use of OC in pig diets, focusing on their effects on redox balance, immune modulation, and production performance. Their use may support sustainable feeding strategies based on locally available agricultural by-products within circular economy systems.

## 1. Introduction

The growing demand for sustainable and efficient pork production has intensified interest in alternative feed ingredients that not only provide essential nutrients but also support animal health and production performance [1,2]. Modern swine production systems are currently facing multiple global challenges, including increasing feed costs, fluctuating availability of high-quality protein sources, environmental constraints, and growing societal expectations regarding sustainable livestock production. In particular, the rising costs of conventional protein and energy feed ingredients, together with the high dependence on imported feed resources in many regions, have created strong pressure to identify alternative and locally available protein and energy sources for pig diets [3,4]. At the same time, the livestock sector is increasingly expected to reduce its environmental footprint and improve resource efficiency [5,6]. This has led to a growing emphasis on circular economy approaches and the utilization of agro-industrial by-products as feed components. In this context, oilseed cakes (OC), produced as by-products of oil extraction from crops such as rapeseed, soybean, sunflower, flax, camelina, cotton, pumpkin and other represent a promising resource. These co-products are rich in protein, residual lipids, fiber, and a range of bioactive compounds, including polyphenols, tocopherols, flavonoids, and phytosterols, which may exert beneficial effects on oxidative balance, immune function, and growth performance in pigs [7,8]. Consequently, OC are increasingly considered not only as alternative protein and energy sources, but also as potential functional feed ingredients in modern swine nutrition. Despite the increasing body of research on oilseed by-products, current studies remain fragmented. Most investigations focus either on their chemical composition, digestibility, and nutritional value, or on their effects on selected production parameters such as growth rate, feed efficiency, or carcass characteristics [9,10]. In contrast, research exploring the biological activity of bioactive compounds present in oilseed by-products often focuses on isolated compounds and is frequently conducted in experimental models other than pigs, such as poultry, rodents, or in vitro systems [11,12]. As a result, the existing literature is dispersed across several research areas—including animal nutrition, physiology, immunology, and redox biology—which limits the development of integrated feeding strategies capable of exploiting the full functional potential of these feed materials. Another important limitation is that the effects of OC on oxidative stress and immune modulation in pigs are still insufficiently characterized. Oxidative stress is a critical factor influencing metabolic efficiency, growth performance, health status, and meat quality in intensive production systems [13], while immune competence directly affects disease resistance, animal welfare, and overall productivity [14]. Recent studies suggest that dietary inclusion of oilseed by-products rich in bioactive compounds may improve antioxidant defense mechanisms, enhance the activity of antioxidant enzymes, reduce markers of oxidative damage, and modulate immune responses [2,3,15]. However, these physiological responses are rarely evaluated together with production performance indicators such as average daily gain (ADG), feed conversion ratio (FCR), or nutrient utilization efficiency [15,16]. Moreover, emerging evidence indicates that hemp-derived co-products may represent a promising source of bioactive compounds with antioxidant potential, although data in swine remain limited and require further investigation [17,18].

Therefore, there is a clear need for comprehensive and integrative analyses that combine information on the occurrence and bioavailability of bioactive compounds in OC, their potential influence on redox balance and immune function, and their subsequent effects on growth performance and health in pigs. Such an approach would help consolidate currently dispersed knowledge and provide a more holistic understanding of the functional role of oilseed by-products in swine nutrition. Furthermore, the effective utilization of these feed materials may contribute to more sustainable pig production systems by reducing dependence on imported protein and energy sources, increasing the use of locally available feed resources, and supporting circular economy principles through the valorization of agro-industrial by-products while simultaneously addressing environmental, economic, and animal welfare objectives [7,19].

## 2. Materials and Methods

### Literature Search Strategy

This manuscript was prepared as a narrative literature review, aiming to provide a comprehensive overview of current knowledge regarding the use of oilseed cakes (OC) as feed ingredients in swine nutrition, with particular emphasis on their effects on oxidative status, immune function, growth performance, carcass characteristics, and meat quality. To ensure broad coverage of the available evidence, a structured literature search was conducted using the Web of Science Core Collection, Scopus, PubMed, and Google Scholar databases. Publications published between 2000 and May 2026 were considered, allowing the inclusion of both pioneering studies and the most recent advances in this field. The search strategy combined keywords related to oilseed processing by-products, pig nutrition, oxidative stress, immune response, growth performance, and carcass quality using Boolean operators (AND, OR). Representative search terms included: oilseed cake, rapeseed cake, soybean cake, sunflower cake, camelina cake, milk thistle cake, walnut cake, flaxseed cake, hemp cake, poppy seed cake, grape seed cake/pomace, olive cake, gardenia cake, Jatropha cake. Equivalent search syntax was adapted to the requirements of individual databases. Searches were performed within the title, abstract, and keyword fields whenever database functionality permitted. In Google Scholar, only the first 200 records sorted by relevance were screened because of the large number of retrieved records and the declining relevance of subsequent results.

Studies were considered eligible if they met one or more of the following criteria:

- Published in peer-reviewed scientific journals and written in English;
- Investigated OC or mechanically pressed oilseed by-products intended for animal nutrition;
- Reported original in vivo experimental studies evaluating the effects of OC on pigs, including growth performance, nutrient digestibility, oxidative status, antioxidant defense, immune responses, gut health, carcass characteristics, meat quality, or other physiological parameters;
- Reported in vitro, analytical, or laboratory-based studies describing the chemical composition, nutrient profile, bioactive compounds, anti-nutritional factors, technological properties, or processing characteristics of OC, provided that these data contributed to the characterization of the feed materials discussed in the review;
- Provided sufficient methodological information to allow interpretation of the experimental findings.

The following publications were excluded:

- Review articles, conference abstracts, book chapters, patents, editorials, and non-peer-reviewed publications (except where reviews were cited to provide general background information);
- Studies conducted on animal species other than pigs when evaluating production, physiological, antioxidant, immune, carcass, or meat quality responses;
- In vitro studies investigating biological responses unrelated to the characterization of OC (e.g., cell culture experiments evaluating pig physiology or immune function) without relevance to feed ingredient composition or functional properties;
- Studies evaluating solvent-extracted oilseed meals without sufficient information to distinguish them from mechanically pressed OC;
- Publications lacking adequate methodological information or reporting duplicate datasets.

Publications were selected according to their scientific relevance to the scope of this review. Preference was given to original peer-reviewed experimental studies evaluating the nutritional and functional effects of OC in pigs. Review articles were used primarily to provide background information and to support the interpretation of experimental findings. Reference lists of relevant publications were additionally screened to identify further studies not retrieved during the electronic database search. During literature evaluation, particular attention was paid to the terminology used for oilseed processing by-products terms such as oilseed cake, expeller cake, cake meal, and oilseed meal are frequently used interchangeably in the literature despite differences in processing technology. Therefore, whenever sufficient methodological information was available, the production method (mechanical cold- or hot-pressing versus solvent extraction) was verified to ensure accurate classification of the feed ingredients included in this review. This approach enabled a more accurate interpretation and comparison of studies involving mechanically pressed OC and other oilseed-derived feed materials. Titles and abstracts were independently screened by three authors, followed by full-text evaluation of potentially eligible publications. Any disagreements regarding study eligibility were resolved through discussion until consensus was reached. In addition, the reference lists of all eligible articles were manually searched to identify relevant publications that were not retrieved during the electronic database search. As this study was designed as a narrative literature review, rather than a formal systematic review, no PRISMA protocol, risk-of-bias assessment, or formal methodological quality appraisal was performed. The objective was to critically synthesize and discuss the available evidence while highlighting current knowledge, practical implications, and future research directions. A conceptual graphical abstract was developed to illustrate the main hypotheses and assumptions of the study, presenting the proposed relationships between OC composition, antinutritional factors, redox regulation, immune modulation, gut health, growth performance, carcass characteristics, and meat quality.

## 3. Types and Nutritional Characteristics of Oilseed Cakes

### 3.1. Chemical Composition

Oilseed cakes can be classified according to the oil extraction method into cold-pressed cakes and hot-pressed cakes. These processing technologies markedly influence the residual oil content, protein concentration, amino acid (AA) availability, and the levels of bioactive and antinutritional compounds [20]. The nutritional value of oilseed by-products is therefore determined not only by the botanical origin of the seed but also by the processing conditions applied during oil extraction [7,20]. Cold-pressed cakes generally retain a considerable proportion of residual oil, typically exceeding 10% of DM and often ranging between 8 and 20%, depending on the oilseed species and pressing efficiency. As a result, they represent valuable sources of both protein and energy, while also supplying substantial amounts of unsaturated fatty acids (UFA), tocopherols, phenolic compounds, and other natural antioxidants [7,21,22]. Cold pressing better preserves thermolabile nutrients and improves the retention of nutritionally valuable compounds [23]. In contrast, hot-pressed cakes are produced from seeds subjected to thermal conditioning prior to mechanical extraction. The application of elevated temperatures increases oil recovery efficiency, resulting in lower residual oil concentrations, typically ranging from 5 to 12%, and consequently higher concentrations of crude protein compared with cold-pressed cakes [20,24]. Thermal treatment may additionally improve nutrient utilization by reducing the activity of certain antinutritional factors, such as trypsin inhibitors. However, excessive heating can impair protein quality through Maillard reactions and reduce the availability of heat-sensitive AA, particularly lysine [25]. Solvent-extracted meals generally contain the lowest residual oil levels and the highest relative protein concentrations, but also reduced amounts of lipid-associated bioactive compounds. The remaining lipids contribute considerably to the energy value of the feed and provide UFA that are important for animal metabolism and physiological functions. Consequently, differences in extraction technology should be carefully considered when evaluating and comparing the nutritional and functional properties of oilseed by-products intended for animal feeding [21,26]. Protein is the dominant component of most OC, and its concentration may reach approx. 56% depending on the oilseed species and processing conditions [1]. Oilseed cakes derived from flaxseed, hempseed, pumpkin, rapeseed and sunflower seeds have been reported to contain between 31.78 and 57.47% crude protein, with the highest concentrations, which are generally observed in finer fraction (<250 µm) obtained after sieving [27]. These finer fractions are also characterized by elevated mineral concentrations, significantly improving the nutritional quality of OC. Due to their favorable AA profiles and high digestibility, OC are considered valuable alternative protein sources for pig nutrition [14,28]. Depending on the botanical origin and processing technology, OCs provide considerable amounts of essential AA, including lysine, methionine, threonine, valine, isoleucine, leucine, phenylalanine, and histidine. Soybean cake is characterized by a high lysine content, while rapeseed and camelina cakes are recognized as good sources of sulfur-containing AA, particularly methionine and cysteine [29]. Sunflower cake contains substantial levels of methionine but is relatively low in lysine, whereas flaxseed cake contributes appreciable amounts of branched-chain AA and sulfur AA [28,30]. Carbohydrates in OC are mainly present as structural polysaccharides, including cellulose and hemicellulose, which constitute the major fraction of dietary fiber [31]. Fiber content may reach up to approx. 66%, depending on the oilseed type and processing conditions [7]. Oilseed cakes are therefore considered rich sources of non-starch polysaccharides that influence the physicochemical characteristics of feed, including water-holding capacity (WHC), swelling properties, and fermentability. In addition, higher carbohydrate and fiber contents are often associated with coarser flour fractions obtained during sieving. Oilseed cakes also contain significant quantities of minerals, usually expressed as ash content, which generally ranges from approx. 5% to over 11% depending on the species and analyzed fraction [15]. These minerals originate directly from the seeds and contribute to the overall nutritional value of OC used in animal feeding. Flaxseed oilcake, for example, has been reported as a nutrient-rich functional ingredient. The chemical composition of OC is therefore highly variable and influenced by multiple factors, including oilseed genotype, cultivation conditions, oil extraction technology and subsequent processing methods. Understanding these compositional differences is essential for optimizing the use of oilseed cakes in swine nutrition and maximizing their nutritional and technological value [32]. Considerable discrepancies among published studies may also result from differences in dietary inclusion levels. While moderate dietary supplementation frequently improves antioxidant status, immune function, or meat quality, higher inclusion rates may increase dietary fiber intake and the concentration of anti-nutritional compounds, thereby reducing nutrient digestibility and growth performance. Consequently, beneficial responses observed at one inclusion level cannot necessarily be extrapolated to higher dietary concentrations. 

Furthermore, physiological stage represents another important source of variability. Weaned piglets generally exhibit greater sensitivity to oxidative stress, intestinal dysfunction, and immune challenges than growing-finishing pigs or sows. Therefore, bioactive compounds present in OC often exert more pronounced effects in young pigs, whereas responses in finishing pigs are more frequently associated with carcass composition, lipid metabolism, and meat quality. Differences in experimental duration, diet formulation, genetics, housing conditions, and health status further contribute to the inconsistent findings reported across studies. Overall, the available evidence indicates that the biological effects of OC cannot be attributed solely to the oilseed species. Instead, they result from complex interactions among botanical origin, processing technology, residual oil content, concentrations of bioactive and anti-nutritional compounds, dietary inclusion level, and the physiological status of the animals [33]. These factors should therefore be carefully considered when comparing studies and formulating practical recommendations for the use of OC in pig nutrition.

### 3.2. Functional and Bioactive Compounds

Oilseed cake contains not only valuable sources of protein and energy in swine nutrition, but also rich sources of numerous functional and bioactive compounds that may positively influence animal health, oxidative status, immune function, and production efficiency. As shown in Table 1, individual OC differ considerably in their profile of bioactive compounds which determines their antioxidant, anti-inflammatory, immunomodulatory, and metabolic properties. In recent years, increasing attention has been focused on the use of oilseed by-products as natural feed additives capable of improving animal health while reducing the need for synthetic additives and antibiotic growth promoters.

Soybeans are a common ingredient of animal feed. They contain isoflavones, which are known to act as phytoestrogens in animals [34]. Soybean products are also rich in triterpenoid saponins and sinapic acid, compounds associated with immunomodulatory and antioxidative effects [35]. These substances may contribute to improved intestinal barrier integrity, reduced inflammatory processes, and enhanced antioxidant protection through scavenging reactive oxygen species.

Rapeseed cake contains glucosinolates which are traditionally regraded as antinutritional compounds due to their potential negative effects on thyroid function and feed intake (FI). However, at moderate dietary levels, these compounds may also participate in detoxification processes and activation of cellular defense mechanisms [36]. Rapeseed cake is also a source of sinapine, a phenolic compound with strong antioxidant properties and the ability to limit lipid peroxidation [37]. 

Sunflower cake is recognized as a valuable source of phenolic compounds, particularly chlorogenic acid and total polyphenols, which exhibit strong antioxidant activity [38,39]. These compounds may reduce lipid oxidation, improve oxidative stability of tissues, and positively influence gut microbiota and intestinal barrier function, thereby supporting immune homeostasis.

Pumpkin seed cake contains phytosterols and tocopherols with important biological functions. Phytosterols are involved in lipid metabolism regulation and exhibit hypolipidemic activity, whereas tocopherols act as natural antioxidants protecting cell membranes against oxidative damage [40,41]. The presence of these compounds may positively affect meat quality and oxidative stability of intramuscular fat. Evening primrose cake is characterized by the presence of γ-linolenic acid (GLA), a precursor of anti-inflammatory eicosanoids involved in immune response regulation [42]. This by-product is also distinguished by a very high antioxidant potential resulting from the presence of numerous polyphenols and phenolic acids. Peschel et al. [42] demonstrated that evening primrose cake exhibited antioxidant activity comparable to commercial green tea and grape seed cake. The high biological activity of this product is probably associated with the presence of catechins, proanthocyanidins, epicatechin derivatives, and other phenolic compounds capable of scavenging free radicals and protecting lipids against oxidation. Black cumin cake represents a valuable functional feed component due to its high content of biologically active compounds, particularly thymoquinone, which is considered the principal constituent responsible for the health-promoting properties of this plant [43,44]. Black cumin seeds also contain alkaloids, saponins, flavonoids, UFA, and phenolic compounds exhibiting antioxidant, anti-inflammatory, and antimicrobial activities [43]. Numerous studies have demonstrated that compounds present in *Nigella sativa* may reduce oxidative stress through free radical scavenging, inhibition of lipid peroxidation, and enhancement of antioxidant enzyme activity, including catalase (CAT) and superoxide dismutase (SOD). Moreover, thymoquinone may modulate immune responses by influencing pro-inflammatory cytokines and the activity of immune cells [38]. Black cumin has also been reported to exhibit broad antimicrobial activity against Gram-positive and Gram-negative bacteria, fungi, viruses, and parasites. Particularly strong activity has been observed against *Staphylococcus* spp., *Escherichia coli*, and *Pseudomonas aeruginosa* [43]. Additionally, studies indicate hepatoprotective effects and beneficial influences on gastrointestinal function and nutrient utilization in animals [45]. Milk thistle cake is a rich source of silymarin and flavonolignans, including silibinin, isosilibinin, silydianin, and silychristin. These compounds exhibit antioxidant and hepatoprotective properties [46]. Bedrníček et al. [47] emphasized that milk thistle cake may serve as a valuable source of functional compounds supporting liver function and detoxification processes. Due to their antioxidant activity, flavonolignans may also reduce oxidative stress associated with intensive swine production systems. Walnut cake contains polyphenols and phytosterols with strong antioxidant and lipid regulating properties. Pycia et al. [48] demonstrated that walnut products are characterized by high concentrations of polyphenolic compounds, particularly ellagitannins, responsible for their strong antioxidant activity. The presence of phytosterols and polyunsaturated fatty acids (PUFA) may additionally improve lipid metabolism and fat quality in animal tissues. Flaxseed cake is recognized as a valuable source of lignans exhibiting antioxidant activity and hormonal modulation effects [49]. Flax products also contain mucilage with prebiotic properties that may support beneficial intestinal microbiota and improve gut health. Furthermore, flaxseed belongs to the richest plant sources of phytosterols among oilseeds [34]. Poppy seed cake is another valuable oilseed by-product rich in protein, dietary fiber, tocopherols, minerals, and PUFA. Muhizi and Kim [50] demonstrated that dietary supplementation with poppy seed cake improved growth performance, feed utilization, and dry matter (DM) digestibility in pigs. Importantly, no negative effects on hematological parameters or fecal microbiota composition were observed, indicating good tolerance of this feed component by animals. The beneficial effects of poppy seed cake may be associated with the presence of tocopherols and other antioxidant compounds improving oxidative stability and reducing oxidative stress. 

Hempseed cake contains unique lignanamides (cannabisins), phenyl propionamides, and catechins exhibiting antioxidant and anti-inflammatory properties [50]. Hemp-derived products are also rich in PUFA, particularly α-linolenic acid (ALA), stearidonic acid, and GLA. Vodolazska and Lauridsen [51] demonstrated that hemp oil supplementation influenced the FA profile of sow milk and piglet plasma, increasing the concentration of long-chain n-3 FA in young animals. Hemp products are also a source of tocopherols, particularly γ-tocopherol, which protects PUFA against oxidation and supports oxidative stability of tissues. Although numerous pieces of information cited above indicate that OC contain compounds with documented biological activity—including soybean isoflavones and saponins, rapeseed sinapine, sunflower chlorogenic acid, pumpkin phytosterols and tocopherols, evening primrose γ-linolenic acid, *Nigella sativa* thymoquinone, milk thistle silymarin, walnut polyphenols, flax lignans, hemp lignanamides, and poppy tocopherols—the magnitude of their effects varies considerably among studies. Such inconsistencies are likely attributable not only to differences in oilseed species but also to variation in processing technology, dietary formulation, inclusion level, animal genotype and age, health status, and experimental design. Therefore, direct comparisons between studies should be interpreted with caution, and future research should focus on standardizing the characterization of oilseed cakes and identifying optimal inclusion strategies for specific categories of pigs.

**Table 1:** Bioactive compounds in selected oilseed cakes used in animal nutrition.

Oilseed Cake	Bioactive Compound	Content (Range)	Unit	Biological Function	References
Soybean	Isoflavones (genistein, daidzein)	1.7–2.9 0.014–0.021 mg/100 g	mg/g oil cake mg/g seed	Antioxidant, phytoestrogenic	[52,53]
	Saponins	2–6	mg/g seed	Immunomodulatory, antioxidant	[52,54]
Rape	Glucosinolates	9.8–14.0	µmol/g cake (DM basis	Antinutritional, detoxification-related	[55,56,57]
	Sinapine	0.0042–0.0070	mg g cake (free phenolic)	Antioxidant	[58,59]
	Sinapic acid	0.029–0.044	mg/g press cake extract	Antioxidant	[60]
Sunflower	Chlorogenic acid	609–5490	mg/g cake extract	Antioxidant	[61,62,63]
	Total phenols	0.0077 ± 0.116	mg/g cold-pressed sunflower oil	Antioxidant	[64,65]
Pumpkin	Phytosterols	0.0039	mg/g seed cake DM	Hypolipidemic	[66]
	Tocopherols	1.183±0.40	mg/g seed cake dry weight	Antioxidant	[41]
Evening primrose	Polyphenols	171c696.4	mg/g seed cake extract (dry extract)	Antioxidant	[42]
Black cumin	Thymoquinone	0.13–0.57	mg/g oil extracted from seed cake	Antimicrobial, antioxidant	[67]
Milk thistle	Flavonolignans including Silymarin	13.62–51.93	mg/g oilseed cake flour (DM)	Hepatoprotective Antioxidant	[46]
Walnut	Polyphenols	41.39–230.45	mg/g oil cake extract	Antioxidant	[68]
	Total phenolic content	2.2–14.8 mg	mg/g oil cake	Antioxidant, anti-inflammatory, lipid metabolism	[69]
Flax	Phenolic acid, flavonoids, and lignans	2–24	mg/g flaxseed SDG-lignan concentrate (SLE fraction)	Antioxidant, hormonal modulation Prebiotic	[7,48]
Poppy	Tocopherols	0.0469	mg/g cold-pressed cake (fresh matter)	Antioxidant	[70]
Hemp	Phenolic acids	0.136–0.623	mg/g whole ground cake (WHC, DM)	Antioxidant, anti-inflammatory, gut microbiota modulation	[71]
	Flavonols	0.114–0.760	mg/g whole ground cake (WHC, DM)	Antioxidant, immunomodulatory, gut health-promoting	[71]

### 3.3. Antinutritional Factors

Despite their high nutritional value and the presence of numerous bioactive compounds, OC also contain antinutritional substances that may limit their application in monogastric animal nutrition, particularly in swine production systems. These compounds can negatively affect nutrient digestibility, energy utilization, gastrointestinal function, and overall production efficiency. The type and concentration of antinutritional factors vary depending on the plant species, cultivar, environmental conditions, and processing technology applied during oil extraction. The most important antinutritional compounds identified in OC include glucosinolates, protease inhibitors, phytates, tannins, alkaloids, saponins, raffinose-family oligosaccharides, cyanogenic glycosides, and excessive levels of crude fiber [72,73].

One of the most extensively studied antinutritional factors in oilseed by-products are glucosinolates present in rapeseed-derived products. These sulfur-containing secondary metabolites, characteristic of Brassicaceae plants, may undergo enzymatic hydrolysis to form compounds such as isothiocyanates, nitriles, and thiocyanates. These degradation products exhibit goitrogenic activity and may interfere with thyroid metabolism by reducing iodine uptake and disrupting thyroid hormone synthesis. Long-term consumption of excessive glucosinolate levels may contribute to thyroid enlargement, impaired liver and kidney function, reduced FI, lower growth performance, and poorer feed efficiency in pigs. Although modern “double-low” rapeseed cultivars contain substantially reduced concentrations of glucosinolates and erucic acid compared with older varieties, these compounds are still regarded as one of the primary limitations restricting high dietary inclusion of rapeseed cake in swine nutrition [74].

Soybean cake contains several biologically active compounds with antinutritional activity, particularly protease inhibitors such as Kunitz trypsin inhibitor and Bowman–Birk inhibitor. These compounds decrease the activity of digestive proteolytic enzymes, thereby reducing protein digestibility and AA utilization. Prolonged exposure to high levels of trypsin inhibitors may result in pancreatic hypertrophy, increased secretion of digestive enzymes, and impaired FCR. Young animals are considered especially sensitive due to the incomplete development of their digestive system. Soybean products also contain raffinose-family oligosaccharides, mainly raffinose and stachyose, which cannot be hydrolyzed by endogenous digestive enzymes in pigs and therefore undergo microbial fermentation in the large intestine. This process may increase gas production and negatively affect digestive comfort and nutrient utilization [75].

Phytic acid represents another important antinutritional factor widely distributed in OC. Phytates function as phosphorus storage compounds in seeds but simultaneously exhibit strong chelating properties toward minerals such as calcium, zinc, iron, and magnesium. As a consequence, insoluble complexes are formed, reducing mineral bioavailability and limiting their absorption by animals. In addition, phytic acid may interact with proteins and digestive enzymes, decreasing nutrient digestibility and feed utilization efficiency. High concentrations of phytates have been reported in hempseed, flaxseed, pumpkin seed, and sunflower cakes [72,76].

Flaxseed and hempseed cakes additionally contain cyanogenic glycosides capable of releasing hydrogen cyanide following enzymatic hydrolysis. In flaxseed products, linustatin and neolinustatin are considered the most relevant cyanogenic compounds and may interfere with cellular respiration and energy metabolism. Flaxseed by-products also contain condensed tannins and trypsin inhibitors that may further reduce protein digestibility and mineral availability [72]. Hempseed cakes are similarly characterized by the presence of phytates, saponins, cyanogenic glycosides, and protease inhibitors. Moreover, substantial differences in antinutritional compound concentration have been observed among hemp cultivars, indicating the importance of genotype selection for feed production purposes [72,73].

High crude fiber concentration is another major factor limiting the use of OC in swine diets. This issue particularly concerns rapeseed, sunflower, hempseed, and milk thistle cakes. Excessive dietary fiber reduces the metabolisable energy concentration of feed, decreases AA and protein digestibility, and may limit voluntary FI. In the case of milk thistle cake, the high concentration of lignin and insoluble fiber fractions additionally lowers nutrient digestibility despite the presence of valuable bioactive compounds such as silymarin and flavonolignans [77].

Evening primrose cake may also contain compounds negatively affecting feed utilization. Peschel et al. [43] reported considerable antitrypsin activity in evening primrose seed residues, which may reduce protein digestibility and AA utilization in monogastric animals.

Despite its well-documented antioxidant and antimicrobial properties, Nigella sativa cake also contains compounds that may exhibit antinutritional effects when included at high dietary levels. The presence of tannins, phytates, saponins, and certain alkaloids may impair mineral bioavailability and protein digestibility. Considerable variation in antinutritional compound concentration has also been reported among different black cumin genotypes, suggesting that cultivar selection strongly influences the nutritional quality of this by-product [78].

In pumpkin seed and sunflower cakes, the predominant antinutritional compounds include phytates, tannins, and phenolic compounds. Although phenolics possess antioxidant activity, excessive concentrations may reduce protein digestibility through interactions with dietary proteins and digestive enzymes [79,80].

Poppy seed cake may contain trace amounts of opium alkaloids, primarily morphine and codeine, which may exert toxic effects when consumed in excessive amounts. At the same time, poppy-derived products are rich in tocopherols and phenolic antioxidants, making them nutritionally valuable but requiring careful consideration regarding feed safety and dietary inclusion levels [81,82].

Glucosinolates, protease inhibitors, phytates, cyanogenic glycosides, tannins, and excessive fiber are among the most important compounds affecting nutrient utilization and animal performance. Nevertheless, advances in plant breeding, processing technologies, and feed enzyme supplementation have substantially reduced many of these constraints. Overall, the available literature indicates that antinutritional factors remain a key consideration in the utilization of OC as feed ingredients in pig nutrition. However, the traditional view of oilseed cakes as nutritionally constrained by the presence of antinutritional factors is gradually changing due to advances in plant breeding, optimization of processing technologies, and the application of targeted enzyme supplementation strategies. Future research should focus on establishing precise dose–response relationships for individual and combined antinutritional compounds, elucidating potential interactions among different antinutritional factors, and developing standardized methods for assessing the nutritional quality and safety of OC used in animal feeding. Moreover, further investigations are needed to evaluate long-term effects of OC inclusion on animal health, nutrient utilization, gut function, and overall production performance under practical farming conditions. A better understanding of the mechanisms underlying antinutritional factors activity and effective mitigation strategies will enable more accurate formulation of diets and wider use of oilseed cakes as sustainable protein sources. Consequently, the successful incorporation of OC into pig diets requires an integrated approach that considers both their nutritional value and potential limitations.

## 4. Potential Role of Oilseed Cakes in Redox Regulation

### 4.1. Oxidative Stress in Pig Production

Oxidative processes play a crucial role in pigs raised under intensive production conditions, where animals are exposed to numerous stressors, including weaning, transportation, high stocking density, and heat stress. These factors contribute to the excessive generation of reactive oxygen species (ROS), disruption of redox homeostasis, and the development of oxidative stress. Weaning is associated with increased ROS production, mitochondrial dysfunction, and enhanced oxidative damage to lipids and DNA, accompanied by a compensatory activation of antioxidant enzymes such as SOD and glutathione peroxidase (GPx) [83,84]. Heat stress further exacerbates lipid peroxidation and damage to proteins and genetic material [85], whereas transportation and high stocking density promote inflammatory processes and secondary ROS production, negatively affecting pig health and productive performance [86]. Under such conditions, nutrition becomes a particularly important factor capable of modulating the oxidative status of the organism.

### 4.2. Bioactive Compounds in Oilseed Cakes and Their Impact on Oxidative Status in Swine

The OC, which are by-products of oil extraction, represent an important source of protein and energy, but at the same time exert a complex influence on the oxidative-reductive balance. On one hand, they contain substantial amounts of PUFA, which, due to their high susceptibility to oxidation, may increase lipid peroxidation and ROS generation. On the other hand, they are rich in numerous bioactive compounds with antioxidant properties, including polyphenols, tocopherols, lignans, and isoflavones [87]. The biological effects of OC therefore depend on the balance between potentially pro-oxidative components, such as PUFA, and antioxidant compounds capable of regulating oxidative processes [88]. This balance may determine whether a specific OC contributes primarily to oxidative challenges or provides protection against excessive ROS formation. 

The concentration of these compounds varies considerably among plant species. The highest total phenolic content (TPC) has been reported in milk thistle cake (30.44 mg GAE/g DM), which is attributed to the presence of flavonolignans (the silymarin complex), together with high antioxidant activity (ABTS + 86.42 mg AAE/g; 2,2-diphenyl-1-picrylhydrazyl (DPPH) 11.36 mg AAE/g) [89,90,91]. Flaxseed, rapeseed, sunflower, and safflower cakes exhibit moderate antioxidant activity (ABTS+ values of 6.13, 8.47, 17.21, and 6.79 mg AAE/g, respectively), whereas poppy seed and pumpkin cakes show considerably lower activity (approx. 3.02 and 4.34 mg AAE/g, respectively), indicating substantial differences in the redox potential of these feed materials [27]. These differences highlight that the antioxidant potential of OC is strongly dependent on the botanical origin of the raw material and the specific composition of secondary metabolites. Therefore, the nutritional value of OC cannot be assessed solely on the basis of their macronutrient content, but should also include evaluation of their bioactive profile [14].

Considerable variation in TPC, total flavonoid content (TFC), and antioxidant potential has also been reported by Hallouch et al. [88]. These authors analyzed pressed cakes obtained after oil extraction from almond, argan, soybean, sunflower, sesame, and black cumin seeds. The results demonstrated significant differences in the concentration of bioactive compounds among the evaluated OC. Sunflower cake exhibited the highest total phenolic content (TPC; 9.82 ± 0.02 mg GAE/g DM) and total flavonoid content (TFC; 15.44 ± 0.04 mg QE/g DM), suggesting a particularly high antioxidant potential compared with the other by-products investigated. Sunflower cake also showed the highest antioxidant activity as determined by both ferric reducing antioxidant power (FRAP) and DPPH assays. These findings confirm the important contribution of phenolic compounds to the antioxidant capacity of oilseeds and their derived products. The results obtained by Hallouch et al. [88] indicate that sunflower cake showed the highest antioxidant capacity among the evaluated OC under the applied analytical conditions. However, this observation should be interpreted with caution, as in vitro antioxidant assays such as FRAP and DPPH reflect chemical antioxidant capacity but do not directly predict biological effectiveness after ingestion. The biological activity of OC may therefore depend not only on the quantity of phenolic compounds but also on their chemical structure, interactions with other bioactive constituents, and bioavailability within the gastrointestinal tract. Mariod et al. [89] demonstrated that phenolic fractions obtained from black cumin cake exhibited high antioxidant activity, indicating that antioxidant effectiveness of OC is not necessarily associated only with the highest TPC values, but may also result from the presence of specific bioactive compounds with high radical-scavenging capacity. 

Interesting observations regarding the relationship between TPC and TFC in whole sesame seeds and the corresponding pressed cakes were reported by Melo et al. [92]. The authors found that sesame seeds before oil extraction contained 74 mg GAE/100 g fresh matter, whereas the concentration increased to 153 mg GAE/100 g fresh matter in the resulting oil cake. A similar trend was observed for TFC, with values of 74 and 67 mg ECE/100 g fresh matter, respectively. According to the authors, this phenomenon may be explained by the fact that the majority of phenolic compounds remain in the solid residue, while only a fraction is removed with the extracted oil. These findings indicate that oil extraction does not necessarily reduce the biological value of the remaining solid fraction; instead, it may contribute to the enrichment of specific bioactive compounds in the resulting cake. From a nutritional perspective, this suggests that OC may represent concentrated sources of phytochemicals that retain functional properties after industrial processing [20].

From a practical perspective, it is particularly noteworthy that sesame oil cakes retained antioxidant activity comparable to, or even exceeding, that of whole seeds (FRAP: seeds 6.8 mmol FSE/100 g vs. cake 8.3 mmol FSE/100 g). Therefore, differences in antioxidant potential among OC may result not only from botanical variation but also from technological factors affecting the stability and retention of bioactive compounds during processing and storage. However, it should be emphasized that the concentration of these compounds depends not only on seed type and cultivar but also on processing conditions and storage practices [7,92]. Therefore, differences in antioxidant potential among OC may result not only from botanical variation but also from technological factors affecting the stability and retention of bioactive compounds during processing and storage. Furthermore, their bioavailability is additionally influenced by particle size reduction and the structural characteristics of the feed material [27]. Consequently, the presence of antioxidant compounds in OC does not necessarily reflect their biological efficacy, as their effects depend on the extent of release from the feed matrix, absorption, and subsequent metabolism in the animal organism. The antioxidant effects of OC result from both the direct scavenging of ROS and the modulation of cellular signaling pathways. Thus, the biological activity of OC should not be interpreted solely through their chemical antioxidant capacity, but also through their ability to regulate endogenous cellular defense mechanisms. Phenolic compounds, tocopherols, and lignans can activate the nuclear factor erythroid 2-related factor 2 (Nrf2), leading to increased expression of antioxidant enzymes such as SOD, CAT, and GPx) [93,94]. Activation of the Nrf2 pathway suggests that bioactive compounds derived from OC may enhance the intrinsic antioxidant capacity of cells by promoting the expression of protective enzymes rather than acting only as exogenous ROS scavengers. At the same time, these compounds may suppress the activation of the nuclear factor kappa B (NF-κB) pathway, thereby reducing inflammatory responses and secondary ROS production [95]. This interaction between oxidative and inflammatory pathways is particularly relevant because excessive activation of inflammatory signaling can stimulate ROS production, while oxidative stress may further promote inflammatory responses through pathways such as NF-κB, resulting in a self-perpetuating cycle of cellular damage [96]. Therefore, the beneficial effects of OC supplementation may arise from both the antioxidant activity of its bioactive constituents and their ability to modulate molecular pathways involved in oxidative and inflammatory homeostasis [97]. Consequently, the biological effects of OC may depend on their ability to influence both antioxidant defense mechanisms and inflammatory processes, although the magnitude and direction of these effects may vary according to the chemical composition of individual by-products. It has been shown that feeding pigs a diet containing 120 g/kg camelina cake significantly increased the expression of key antioxidant enzymes, including SOD, CAT, and GPx in spleen tissue, while simultaneously reducing the levels of pro-inflammatory cytokines such as interleukin-1β (IL-1β) and tumor necrosis factor-α (TNF-α) [98]. These findings suggest that camelina cake supplementation may enhance antioxidant defense and modulate inflammatory responses in pigs. The increased expression of antioxidant enzymes together with the reduction in pro-inflammatory mediators indicates that the biological effects of camelina cake may be associated with the activity of its bioactive constituents, particularly n-3 polyunsaturated fatty acids and other plant-derived compounds, which can influence oxidative status and immune-related signaling pathways [20]. Moreover, a significant increase in TAC was observed, accompanied by marked upregulation of genes involved in ROS detoxification, including SOD (2.27-fold), CAT (3.29-fold), and GPx (1.66-fold). The observed increase in TAC together with the upregulation of antioxidant-related genes suggests that camelina cake may enhance the endogenous antioxidant capacity of pigs. This indicates that its effects are not limited to the supply of dietary antioxidant compounds but may also involve stimulation of cellular mechanisms responsible for maintaining oxidative homeostasis [20]. Increased expression of inducible nitric oxide synthase (iNOS; 5.18-fold) and endothelial nitric oxide synthase (eNOS; 2.74-fold) suggests that camelina cake supplementation may influence nitric oxide (NO)-related pathways involved in redox regulation, vascular function and cellular signaling [98]. The observed changes may reflect modulation of NO bioavailability, which contributes to vascular homeostasis and immune regulation; however, excessive iNOS activation may promote nitrosative stress under conditions of increased oxidative burden. Therefore, the increased expression of NOS enzymes should be interpreted as a potential adaptive response to dietary modification rather than as a direct indicator of either beneficial or adverse effects, since the biological outcome depends on the cellular context, oxidative status and the balance between NO production and antioxidant defense [99]. Other studies have demonstrated that dietary supplementation with olive cake extract effectively alleviated the adverse effects of lipopolysaccharide (LPS)-induced oxidative stress in piglets. LPS challenge was associated with a marked reduction in the activity of antioxidant enzymes, including SOD (2.47-fold) and CAT (3.43-fold) and GPx (1.83-fold), as well as increased concentrations of oxidative damage markers such as malondialdehyde (MDA) and nitric oxide (NO). LPS challenge resulted in impaired antioxidant defense, as reflected by reduced activity of antioxidant enzymes, together with increased accumulation of oxidative damage markers. This confirms that the experimental model effectively induced oxidative and inflammatory imbalance. Supplementation with olive cake extract significantly enhanced SOD and GPx activities while reducing MDA and NO concentrations compared with the LPS-treated control group. The observed restoration of SOD and GPx activities suggests that olive cake-derived compounds may counteract the suppression of antioxidant defenses caused by inflammatory stress. Therefore, the effect of olive cake extract may involve maintaining the functional capacity of enzymatic antioxidant systems during conditions associated with excessive ROS generation [97]. These findings indicate that the bioactive compounds present in olive cake extract strengthen endogenous antioxidant defense mechanisms, thereby limiting lipid peroxidation and cellular damage. The reduction in MDA concentration provides additional evidence that olive cake supplementation effectively decreased oxidative damage at the cellular level. Since MDA reflects the extent of lipid peroxidation, its reduction indicates improved protection of membrane lipids against ROS-mediated degradation [100]. This conclusion is further supported by the reduced activities of lactate dehydrogenase (LDH), alanine aminotransferase (ALT), and aspartate aminotransferase (AST), which are commonly recognized indicators of tissue injury [101]. The reduced activities of LDH, ALT and AST suggest that the antioxidant effects of olive cake extract were associated with attenuation of tissue injury and preservation of cellular membrane integrity. As oxidative stress contributes to membrane lipid peroxidation and increased membrane permeability, limiting oxidative damage may reduce the leakage of intracellular enzymes into the circulation [102]. Therefore, olive cake supplementation may protect tissues not only by improving redox balance but also by alleviating secondary damage associated with inflammation-induced oxidative stress [100,101]. The beneficial effects of bioactive feed components have also been confirmed for grape seed cake inclusion at 50 g/kg of the diet. Such supplementation reduced lipid peroxidation markers, including thiobarbituric acid reactive substances (TBARS), and decreased the concentrations of pro-inflammatory cytokines (IL-1β and TNF-α) in the liver, despite the absence of significant changes in antioxidant enzyme activity [103]. The absence of changes in classical antioxidant enzyme activities, despite reduced lipid peroxidation and inflammatory markers, suggests that grape seed cake may exert protective effects primarily through the activity of its phenolic constituents rather than through stimulation of endogenous antioxidant enzymes. Grape-derived polyphenols, particularly proanthocyanidins, may directly reduce oxidative damage and modulate inflammatory responses [96,100].

These results suggest that the mechanisms of action of polyphenols present in oilseed by-products may involve direct free-radical scavenging and modulation of signaling pathways associated with inflammatory responses, independently of classical antioxidant enzyme systems. This finding highlights that evaluation of OC antioxidant potential should not be limited only to measurements of SOD, CAT, or GPx activity. The biological effects of these by-products may also depend on non-enzymatic mechanisms, including regulation of inflammatory processes and limitation of secondary ROS formation.

Furthermore, supplementation with gardenia seed cake at inclusion levels of 50 and 100 g/kg diet increased SOD activity in the liver and spleen, as well as GSH-Px activity in the longissimus dorsi muscle and spleen. These effects were accompanied by a significant reduction in MDA) concentrations in the analyzed tissues (*p* < 0.05), confirming the effectiveness of this by-product in mitigating oxidative stress and protecting cellular structures against oxidative damage [103]. In contrast to grape seed cake, gardenia seed cake appears to enhance enzymatic antioxidant defense by increasing the activity of key antioxidant enzymes together with reducing lipid peroxidation. The simultaneous increase in SOD and GSH-Px activity and decrease in MDA concentration suggest improved efficiency of endogenous antioxidant protection and reduced oxidative injury in multiple tissues. Collectively, these findings indicate that various OC can effectively modulate both enzymatic and non-enzymatic antioxidant defense mechanisms, thereby contributing to improved health status and physiological functioning in pigs. The presented studies demonstrate that the antioxidant effects of OC are highly heterogeneous and depend on the specific composition of each by-product. Some OC primarily enhance enzymatic antioxidant defense, whereas others exert effects through direct radical scavenging or modulation of inflammatory pathways. Therefore, the biological value of OC should be considered as a result of multiple complementary mechanisms rather than a single antioxidant parameter.

### 4.3. Effects of Oilseed Cakes on the Immune System of Pigs

Under intensive production conditions, the porcine immune system is continuously influenced by environmental and nutritional factors, with diet representing one of the major modulators of immune function. Oilseed cakes, as by-products of oil extraction, constitute valuable feed ingredients with immunomodulatory potential resulting from both their lipid composition and their content of numerous bioactive compounds. Their immunomodulatory activity is associated with the presence of polyunsaturated fatty acids and phytochemicals, including phenolic compounds, lignans, and tocopherols, which may influence inflammatory processes and immune-related signaling pathways. These effects may involve modulation of cytokine production and regulation of signaling pathways associated with immune responses, including NF-κB and PPARγ [104]. Through inhibition of NF-κB activation and stimulation of PPARγ-mediated responses, bioactive dietary compounds may reduce the expression of pro-inflammatory mediators, contributing to improved immune regulation [105]. Particularly relevant in this context is the presence of bioactive compounds including phenolic compounds and lipid constituents, which may influence immune function by modulating inflammatory mediator production and immune cell activity. Phenolic compounds from olive-derived products have been shown to exert anti-inflammatory effects through the regulation of key signaling pathways, including inhibition of NF-κB activation and modulation of cytokine expression [97,106]. In vivo studies demonstrated that a diet containing 120 g/kg camelina cake significantly reduced the expression of pro-inflammatory cytokines, including TNF-α, IL-1β, IL-6, and IL-8 (*p* < 0.05), at both mRNA and protein levels in the spleen. These results indicate that camelina cake supplementation may attenuate excessive inflammatory activation at both transcriptional and functional levels, suggesting a broad effect on immune regulation rather than a response limited to changes in individual cytokines. Simultaneously, it increased the expression of the anti-inflammatory cytokine IL-4 (2.32-fold in the spleen) and elevated its plasma concentration [98]. No significant changes were observed in interferon-γ (IFN-γ) expression, suggesting a selective modulation of the Th1/Th2 balance. The lack of changes in IFN-γ expression suggests that camelina cake did not induce a generalized suppression of immune activity but rather modified the profile of immune responses by shifting the balance toward a less inflammatory state. Similar effects were observed in studies using flaxseed supplementation, where inclusion of 100 g/kg flaxseed linearly decreased the expression of pro-inflammatory cytokine genes in muscle, spleen, and adipose tissue [104]. The consistency of these observations across different plant-derived lipid sources suggests that the immunomodulatory effects may be partly related to the presence of PUFA, particularly their ability to regulate inflammatory signaling pathways.

The immunomodulatory properties of OC are associated with the effects of PUFA and bioactive compounds on intracellular signaling pathways. In the case of camelina cake, the observed reduction in cyclooxygenase-2 (COX-2) expression (*p* < 0.00001), together with decreased activity of NF-κB and MAPK-p38α pathways (1.41- and 3.83-fold reductions, respectively; *p* < 0.02) and increased PPARγ expression (3.53-fold; *p* < 0.0001), provides molecular evidence supporting the anti-inflammatory potential of this by-product [98]. Polyunsaturated FA can directly interact with transcription factors regulating inflammatory responses, thereby suppressing the transcription of pro-inflammatory genes and reducing cytokine production, which constitutes a key mechanism underlying their immunomodulatory effects [107]. Therefore, the effects observed following OC supplementation may result from the combined action of lipid-derived mediators and plant bioactive compounds, which together influence inflammatory signaling, cytokine secretion, and immune cell function [97]. In addition to camelina cake, other oil-processing by-products have also been shown to exert significant effects on immune function in pigs. For example, olive cake extract supplementation alleviated the detrimental consequences of LPS-induced inflammation in piglets. These findings indicate that the biological effects of oil-processing by-products are not limited to the modulation of systemic inflammatory responses but may also involve the protection of intestinal tissues, which represent a key component of immune regulation in pigs. The intestinal barrier plays a crucial role in maintaining immune homeostasis by regulating interactions between dietary components, microbiota and host immune cells, while bioactive compounds derived from plant by-products may support intestinal integrity and attenuate inflammatory responses [108]. Reduced concentrations of the pro-inflammatory cytokines TNF-α and IL-6, together with increased levels of the anti-inflammatory cytokine IL-10, were observed, indicating attenuation of the LPS-induced inflammatory response and a shift toward a more balanced cytokine profile. Moreover, olive cake extract improved intestinal barrier integrity, as evidenced by reduced concentrations of intestinal damage markers (DAO and D-xylose) and improved intestinal morphology, reflected by an increased villus height-to-crypt depth ratio. These changes suggest that olive cake extract may contribute to maintaining intestinal homeostasis by limiting epithelial damage and supporting structural recovery of the intestinal mucosa. Beneficial shifts in gut microbiota composition were also reported, including increased relative abundance of *Lactobacillus* and *Clostridium* species, which may further support local immune mechanisms and gastrointestinal homeostasis [97]. The observed microbiota alterations suggest that the immunomodulatory effects of olive cake extract may be mediated through microbiota–host interactions. Bioactive compounds present in olive cake may promote beneficial microbial populations and microbial metabolite production, such as short-chain fatty acids (SCFAs), which enhance intestinal barrier integrity and regulate mucosal immune responses [109]. The immunomodulatory activity of OC may also be closely linked to their antioxidant properties. Studies have demonstrated that camelina cake, rich in n-3 PUFA and phenolic compounds, simultaneously reduces the expression of pro-inflammatory cytokines (IL-1β, TNF-α, and IL-6) while increasing the activity of antioxidant enzymes such as CAT and SOD, highlighting the close interaction between immune regulation and oxidative stress control [98]. These findings suggest that the beneficial effects of camelina cake may result from the coordinated regulation of inflammatory and oxidative pathways rather than from a single mechanism of action. Similarly, grape seed cake reduced the expression of pro-inflammatory cytokines (IL-1β, IL-8, and TNF-α) and inhibited NF-κB activation in the liver, despite having no significant effect on antioxidant enzyme activity, suggesting a more targeted anti-inflammatory mode of action [110]. The lack of changes in antioxidant enzyme activity, together with reduced inflammatory markers, indicates that the effects of grape seed cake may involve non-enzymatic antioxidant mechanisms or direct modulation of inflammatory signaling pathways. Regarding other plant-derived by-products, supplementation with gardenia cake at 50 g/kg diet significantly reduced plasma concentrations of IL-2 and IL-8 (*p* < 0.05), indicating anti-inflammatory activity, whereas a higher inclusion level (100 g/kg diet) increased immunoglobulin A (IgA) concentrations (*p* < 0.05), suggesting stimulation of humoral immunity [103]. These results indicate that the biological response to OC may depend not only on their chemical composition but also on the inclusion level, with different doses potentially affecting distinct components of immune regulation. These findings indicate that the immunomodulatory effects of OC may be dose-dependent. Furthermore, dietary inclusion of 50 g/kg fermented Jatropha curcas cake modulated immune responses by reducing IL-6 concentrations in the jejunum and colon. Simultaneously, reduced IL-1 levels were observed in the hypothalamus, together with alterations in plasma concentrations of IL-1, IL-6, and IL-10, indicating a systemic immunomodulatory effect. These findings suggest that fermented Jatropha curcas cake may influence immune regulation at both local intestinal and systemic levels. The changes observed in different tissues indicate that the effects of this by-product may extend beyond the gastrointestinal tract and involve broader regulation of inflammatory mediators [109]. These changes were accompanied by beneficial modifications in gut microbiota composition, including increased populations of beneficial microorganisms and elevated concentrations of neuroactive metabolites such as γ-aminobutyric acid and serotonin (5-hydroxytryptamine, 5-HT). The simultaneous changes in microbiota composition and neuroactive metabolites suggest a possible interaction between dietary supplementation, intestinal microorganisms and host signaling pathways. Microbiota-derived metabolites may act as mediators of gut–host communication, although their specific contribution to immune modulation requires further investigation [109]. These observations suggest that dietary fermented Jatropha curcas cake may also influence the gut–brain axis, extending its potential benefits beyond traditional nutritional and production-related outcomes [111]. Therefore, fermented Jatropha curcas cake represents an example of an OC whose biological effects may involve not only direct regulation of inflammatory responses but also interactions within the gut–microbiota–host axis. An important factor determining the efficacy of OC is technological processing, particularly fermentation, which can reduce antinutritional compounds (e.g., glucosinolates in rapeseed cake) while enhancing the bioavailability of bioactive constituents. The biological value of OC is therefore not determined exclusively by their original chemical composition but may also depend on processing methods that modify the availability and activity of their functional components. Such modifications may enhance the utilization of OC in animal nutrition by reducing factors limiting nutrient availability and increasing the accessibility of compounds with potential biological activity [112].

Nevertheless, it should be emphasized that the presence of antinutritional factors may, in some cases, limit the beneficial immunological and metabolic effects associated with these feed materials [20,113]. Therefore, the application of OC in pig nutrition requires careful consideration of both their functional potential and possible limitations related to species-specific composition, processing conditions, and the presence of antinutritional compounds [114].

The effects of various OC on redox balance and immune responses in pigs are summarized in Table 2.

## 5. Effects on Growth Performance and Production Parameters

Oilseed cakes are widely recognized as valuable feed ingredients in pig nutrition, primarily due to their high protein and residual energy content. They are increasingly used as alternative protein sources replacing soybean meal and part of oil and may simultaneously provide bioactive compounds that influence animal performance. However, their nutritional value and production effects depend on oilseed species, processing technology, inclusion level, and the presence of antinutritional compounds. Consequently, responses in pigs may vary from improved growth performance and feed efficiency to reductions in nutrient utilization when excessive dietary inclusion levels are applied.

### Feed Intake, Growth, Feed Conversion Ratio, Carcass Traits and Meat Quality

The OC are a natural solution in the nutrition of many animal species, including pigs, but it has also been repeatedly emphasized that the type of cake, as well as processing methods and inclusion levels, influence a number of production factors during animal fattening. In addition to dietary inclusion level, the processing technology used for OC production represents another important factor influencing its nutritional value and biological activity. Cold-pressed cakes generally retain higher amounts of residual oil, unsaturated fatty acids, tocopherols, and phenolic compounds, thereby providing greater antioxidant potential [7,20,21,22,23]. In contrast, hot pressing increases oil extraction efficiency and protein concentration but may reduce the availability of heat-sensitive amino acids and bioactive compounds due to thermal degradation and Maillard reactions [20,21,22]. Solvent-extracted meals are characterized by the lowest residual oil content and the highest relative protein concentration, although this process also decreases the content of lipid-associated bioactive compounds [15,20]. Furthermore, technological treatments such as fermentation and sieving may improve the nutritional quality of OC by reducing antinutritional factors, modifying fiber characteristics, and increasing the concentration and bioavailability of proteins and selected bioactive compounds [27,115,116]. Therefore, differences in processing technology should be considered when comparing the results of individual studies, as they may partly explain the variability observed in antioxidant status, immune responses, nutrient digestibility, and growth performance in pigs. Despite the existence of many types of OC derived from different oilseeds, their versatility in use is noteworthy, for example, in combination with other animal feed ingredients. It is generally accepted that oil cake can be a high-protein raw material and also contains large amounts of energy, thus playing an important role in mixtures intended for the fattening period of pigs. However, as indicated in the literature, the specified percentage of olive cake in a feed mixture should not be exceeded, as this can negatively impact animal performance; for example, excessive rapeseed cake can negatively impact the amount of feed consumed by animals. As is known, soybean, canola, camelina, and flaxseed co-products contain anti-nutritional factors that can limit dietary nutrient utilization. In their study, Johannsen et al. [116] assumed that a starter diet high in protein derived from fermented soybean cake fed to weaned pigs from organic free-range farming would improve their growth and development during the first 14 days after weaning. Furthermore, the beneficial effect of this feed mixture was assumed to persist after the diet was discontinued. The study also confirmed that increased protein content in the diet would not increase the risk of developing adverse gastrointestinal diseases in weaned pigs from free-range farming due to the addition of fermented soybean cake, which is a very beneficial phenomenon. The conclusions were that pigs fed a control starter diet had a 3% greater average daily feed intake (ADFI) compared to pigs fed a starter diet with fermented soybean cake. The ADG increased by 80 g/kg and the FCR improved by 120 g/kg when pigs were fed the experimental diet compared to the control diet. Rambu et al. [117] confirmed in their research that the addition of 80 g/kg fermented flaxseed cake in the diet of post-weaned piglets (35 days old) improved body weight gain (BWG), ADG, FCR, and diarrhea score, without affecting ADFI, compared with 80 g/kg unfermented flaxseed cakes. Slightly different results were obtained by Stødkilde et al. [1], who compared the production rates of fattening pigs fed imported feeds supplemented with soybeans and sunflower meal with, among other things, an economically advantageous feed containing fava beans and rapeseed cake. They showed that the experimental diet did not affect daily growth; however, daily FI was similar for both groups: 2.74 kg/pig/day and 2.70 kg/pig/day, respectively. The FCR was slightly lower in the experimental group (2.47 kg feed/kg gain) compared to the control group (2.52 kg feed/kg). However, the study showed that local protein sources f.e. rapeseed cake can replace imported protein such as soybean and sunflower meal in fattening pigs without negatively impacting animal productivity. However, as Dorca-Preda et al. [118] indicate, organic pig farming systems are associated with considerable ammonia losses, nitrate leaching in sow pastures, and dependence on imported protein feed components, such as soybean cake. However, further research seems necessary, and the results are promising. There are also reports that not every OC supplement can be used in pig nutrition, as, for example, camelina may contain antinutritional factors (Matthès and Zubr [119], which can affect pig health and productivity. Therefore, camelina by-products were not often used in pig nutrition. Concerns have been raised about these antinutritional factors, which may contribute to poor health in fattening pigs and decreased production rates. However, a subsequent experiment using weaning-age piglets demonstrated the effect of adding 180 g/kg camelina cake to their diet and confirmed that such an addition at this level did not cause any adverse clinical signs or changes in organ morphology in weaning-age pigs, nor did it cause any symptoms that could indicate any toxicity of the feed additive. Pigs fed the feed the experimental diet with 18% camelina cake weighed approx. 5 kg less than pigs in the control group [120]. However, research by Hilbrands et al. [121] showed that feeding up to 5% camelina cake in a diet based on corn and soybean meal had no negative effect on the growth or carcass characteristics of fattening pigs. As confirmed by other authors, camelina by-products are characterized by low total digestibility and energy content, but high AA digestibility. Camelina cake had a higher energy value than camelina meal, while meal is characterized by lower leucine and cysteine digestibility, which is probably explained by the higher fiber concentration in the case of camelina cake. As shown by Rivero et al. [122], the addition of some OC may result in deterioration of performance indicators due to excessive fiber content in the feed. Compared to other popular protein sources, such as rapeseed or soybean meal, the energy value of camelina by-products was also lower, but the digestibility of individual AA, such as methionine, isoleucine, valine, and arginine, may be comparable. Therefore, further research is necessary to clarify the main factors influencing the nutritional value of camelina co-products fed to fattening pigs [123]. Danilov et al. [124] presented the results of a study on the chemical composition of in-shell pumpkin seed cake and its potential use in fattening pig nutrition. It was found that including pumpkin seed cake in the diets of growing pigsc at 40 g and 70 g of the feed mix had no negative impact on health and production performance, which were comparable for all groups studied. In addition to the commonly used OC, the use of Amarula cake to replace soybean meal in regions where it is primarily imported with cake from plants endemic to the area is noteworthy. Alternative, economical, and locally available feeds can easily meet their protein requirements. Thabethe et al. [125] published a study using Amarula cake, a by-product of Amarula seed oil extraction [126]. Amarula fruit is harvested in this region for brewing beer, and the fruit pulp is used to produce a traditional liqueur. Seed oil is also used in the cosmetics industry, highlighting the value of using by-products to minimize financial losses. They obtained results confirming that high levels of Amarula cake linearly reduced ADG, FCR, and scaled FI, while simultaneously increasing ADFI. However, the results suggested that high levels of the supplement (150 and 200 g/kg DM) were detrimental to the growth performance of the native Windsnyer breed pigs. The Amarula cake supplement level was set at an optimal level of approx. 100 g/kg DM. Therefore, this ingredient can be added up to this level without affecting the growth parameters of native pigs. However, the study by Mabena et al. [127] specifies the addition of this cake at a slightly higher level, i.e., 150 g/kg DM. In the study by Joven et al. [112], it was found that olive cake can be added up to 100 g/kg in the diets of fattening pigs, improving some aspects of growth and performance. The cited studies also confirm the widely held view that only a strictly defined dose of oilcake, up to a certain level, brings benefits in terms of improved production parameters. When formulating oilcake-based diets for pigs, both feed weight and fat content should be considered. The aforementioned study by Mabena et al. [127] on amarula oilcake found that the use of this type of oilcake in fattening pig diets reduced the availability of lysine and threonine. Furthermore, the content of most essential AA (i.e., histidine, leucine, phenylalanine, threonine, and valine) and some AA (i.e., alanine, aspartic acid, glycine, etc.) was significantly reduced [127]. This experiment demonstrated that the addition of this cake below 150 g/kg can be used as a potential feed in pig diets, partially replacing soybean meal protein without negatively affecting growth, nutrient digestibility, and carcass characteristics. A content above 150 g/kg in pig diets is associated with reduced FI and BWG. However, Smit and Beltranena. Reference [119] reported that dietary addition of the same amount, i.e., 150 g/kg, of camelina cake (a by-product with an oil) caused ADFI, ADG and BWG of growing pigs decreased. Therefore, further work should be conducted to assess the performance of pigs fed diets containing these OC. Importantly, the effect of Amarula oil FA on pig carcasses should also be assessed. Kropiwiec-Domańska et al. [128] also demonstrated that fattening pigs fed a mixture of domestic protein components, i.e., peas, field beans, sunflower meal, and hemp and linseed cake, were characterized by lower daily gains, higher FI, and thus a less favorable FCR. However, it should be emphasized that the cost of feeding these fattening pigs was significantly lower. Based on the a reviewed literature, it should be noted that the addition of OC, as an alternative protein source replacing soy protein, usually has no effect on the studied traits or is beneficial and improves production indicators, but only up to a specific level in the pigs’ feed and the method of its production [129], This dose dependent response is well illustrated by several OC, whereas increasing the inclusion level to 100–150 g/kg reduced ADG, final body weight, hot carcass weight, and dressing percentage [119,120,130]. Similarly, Amarula cake showed the most favorable responses at approx. 100 g/kg, while inclusion levels exceeding 150 g/kg reduced ADG, FCR, nutrient digestibility, and carcass weight [124,126]. In contrast, pumpkin seed cake included at 40–70 g/kg of the diet had no detrimental effects on health, growth performance, or carcass characteristics [123], whereas olive cake improved growth performance and carcass quality when included at up to 100 g/kg of diet [127]. These findings indicate that the optimal inclusion level should be established individually for each type of OC according to its chemical composition, processing method, and physiological stage of pigs. Some studies emphasize the economic aspect of this solution, as, for example, post-extraction soybean meal imported to Poland, which is also derived from GMO plants, is relatively expensive and may negatively impact the profitability of fattening pig production [129]. Despite the promising nutritional and functional properties of OC, several practical limitations should be considered before their widespread implementation in commercial pig production. The chemical composition of OC may vary considerably depending on the oilseed species, cultivar, growing conditions, harvesting practices, and processing technology, resulting in substantial differences in nutrient composition and concentrations of bioactive and antinutritional compounds [7,20,21,22,23]. Moreover, storage conditions may influence lipid oxidation and the stability of bioactive compounds, thereby affecting the nutritional value and shelf life of OC [7,22]. Additional practical constraints include regional availability, fluctuations in production volume, and economic feasibility, which may influence the cost effectiveness of incorporating OC into pig diets. Therefore, successful implementation of these by-products in commercial swine production requires not only optimization of dietary inclusion levels but also careful consideration of raw material quality, processing methods, and local production conditions.

One of the most important factors influencing meat quality during fattening pig production is nutrition. With a properly balanced feed, it is possible to obtain meat of the desired quality, even with a modified FA profile, for example, to promote health. Kropiwiec-Domańska et al. [128] conducted an experiment in which they replaced soybean meal protein (control group) in the fattening pigs’ diet with a mixture of other protein components sourced from domestic crops, i.e., peas, field beans, sunflower meal, and hemp and linseed cake (experimental group). It was found that the carcasses of fattening pigs from the control group were characterized by statistically significantly higher backfat thickness and lower loin eye height and carcass leanness (by nearly 2%). After carcass dissection, it was found that the carcasses of the experimental fattening pigs were characterized by a higher weight of the most commercially valuable primal cuts. This is closely related to the higher leanness achieved in this study group. No adverse effects of dietary modifications on the economic and commercial value of fattening pigs were observed. However, feeding fattening pigs in the experimental group increased their production profitability—the average feeding cost at the end of fattening was approx. 8% lower. In another study, Zhu et al. [130] examined whether increasing the proportion of camelina cake in pig diets would affect carcass characteristics, pork quality, and belly firmness. Fattening pigs were fed diets based on corn-soybean meal with 0 g/kg (control group), 50 g/kg, 10 g/kg, or 150 g/kg camelina cake to replace the corn and soybean meal for 12 weeks. Increasing the proportion of camelina cake in the diet statistically significantly reduced hot carcass weight, dressing percentage, belly weight, and backfat thickness at the 10th rib, and increased the lean meat percentage. However, there was no effect of feed on the percentage loss of carcass weight after chilling, meat pH at 45 min and 24 h post-mortem, water absorption and marbling of pork chops, or belly firmness with increasing the proportion of camelina cake. However, increasing the proportion of camelina cake decreased (*p* < 0.05) the Warner-Bratzler shear force and the color component (a*) of pork chops. Subjective color and overall appearance results over 7 days were less favorable (*p* < 0.05) for pork chops from pigs fed an increased proportion of camelina cake in the diet to 150 g/kg. However, changes in shear strength, subjective color, and overall appearance of pork chops with camelina cake added to the diet were minor. Similar studies were conducted by Smit and Beltranena [119], who examined the addition of camelina cake with residual oil content to the feed of fattening pigs. The oil content was intended to provide additional energy in the diet but could also enrich the pork with n-3 FA. As previously emphasized, limited information is available on feeding pigs with camelina cake, particularly regarding its nutritional safety (toxicity), growth performance, and the effectiveness of enriching pork with n-3 FA. Therefore, the impact of feeding increased camelina cake (12.2% crude fat) in the diets of piglets during the rearing and fattening periods was assessed. A general clinical examination was performed after slaughter, and internal organs were weighed to determine the toxicity of the additive. Samples of liver, backfat, and fat from the belly and jowl were collected for FA analysis. Pigs were slaughtered at approx. 125 kg body weight (BW). Increasing the proportion of camelina cake in the diet extended the time it took for the animals to reach their target BW. Lower carcass weight (*p* < 0.001), slaughter yield (*p* < 0.050), and backfat thickness (*p* < 0.010) were observed after slaughter, but the diet did not significantly affect loin eye height. Increasing the proportion of camelina cake increased the weight of some internal organs, i.e., liver and pancreas (*p* < 0.050), relative to BW, but did not affect the weight of the heart, thyroid, or kidneys. Increasing the proportion of camelina cake in the diet linearly increased the content of n-3 FA, including docosahexaenoic acid, in back fat and fat in the analyzed carcass elements. In summary, feeding pigs with camelina cake at up to 180 g/kg during the rearing phase and 150 g/kg during the growth, development, and fattening phases did not cause clinical signs of toxicity and also enriched the carcass fat reserves with n-3 FA. Although studies indicate that camelina cake is a by-product proposed as an alternative protein source, data on piglets are still limited. Luise et al. [2] presented a study that aimed to evaluate the effect of different doses of camelina cake on soybean meal replacement on the growth, health, and intestinal health of weaned piglets. On day 14 post-weaning, sixty-four piglets were assigned to a standard diet or a diet containing 4 g/kg, 80 g/kg, or 12 g/kg camelina cake. Piglets were weighed weekly. On days 7 and 28, feces were collected for microbiota and polyamines, and blood for analysis of reactive oxygen metabolites (ROM) and thyroxine. On day 28, pigs were slaughtered, organs were weighed, intestinal pH was measured, and the colon was analyzed for volatile FA. The jejunum was used for morphological and gene expression analysis. The addition of camelina cake to piglet diets decreased mean BWG and reduced FI. Camelina cake increased liver weight (*p* < 0.0001) and affected cadaverine (*p* < 0.001). The diet did not affect ROM, thyroxine, intestinal pH, VFA, or morphology. Feeding camelina cake increased the resilience of the gut microbiome and can be evaluated as a potential alternative protein source with dose-dependent limitations on piglet growth performance. Carcass characteristics and pork quality of local pig breeds are rarely documented, therefore this study (Thabethe et al. [131] was conducted to determine the relationship between increasing levels of Amarula cake in the diet of fattening pigs and carcass traits, basic pork cuts, meat quality and relative internal organ weight in the native Windsnyer breed of pigs. Pigs were examined during the growing period when they were approx. 67 days old. The study lasted six weeks. After slaughter, data on carcass traits, basic pork cuts and relative internal organ weight of Windsnyer pigs were analyzed using statistical methods. A negative linear relationship was found between increasing levels of Amarula cake, carcass length, hot and cold carcass weight. Stomach weight, backfat thickness, juice leakage and hepato-somatic index increased linearly with increasing levels of Amarula cake. Heart, lungs and spleen were not associated with increasing levels of Amarula cake. Increased levels of Amarula cake diets impaired carcass characteristics and selected internal organs, therefore, Windsnyer pigs can only be fed Amarula cake up to 100 g per kilogram of DM, as previously demonstrated by these authors.

An experiment was also conducted to determine the effects of adding cottonseed cake to rations for weaned, growing pigs [132]. Thirty-two Landrace x Large White pigs were slaughtered after reaching a live weight of approx. 75.0 kg. The chilled carcasses were then dissected, and the weight of internal organs was recorded. The estimated productivity of the pigs on each diet was calculated. Cottonseed cake statistically significantly reduced voluntary FI and consequently, BWG, and increased the weight of the heart, kidney, and liver. Pigs on the soy-based control diet reached slaughter weight in a shorter time compared to the pigs in the experimental group. This result was likely also partially due to lysine deficiency. It has been found that currently in Cameroon, it is profitable to add cottonseed cake to the diet of weaned piglets at a dose of up to 300 g/kg. The possibility of feeding pigs Amarula cake at a dose of up to 100 g per kilogram of DM has also been confirmed. Another example of the use of native OC species is the study by Arjin et al. [133] on the Perilla plant. Perilla is an edible oil plant containing high levels of PUFA, such as alpha-linolenic acid (ALA) and n-3 FA. In this study, the effect of perilla cake supplementation in pig diets on production performance, carcass traits, meat quality, and FA composition in fat tissue and meat. The slaughter performance of fattening pigs improved in terms of ADG in the experimental group after perilla cake supplementation. However, the quality of the pork carcass and meat remained unchanged. Furthermore, the addition of perilla cake increased the content of PUFA and reduced the proportion of saturated fatty acids (SFA and UFA in pork). The results showed that ADG of fattening pigs significantly increased. However, supplementation did not affect carcass traits or meat quality, except for color brightness. Dietary supplementation significantly increased the level of α-linolenic acid (ALA, C18:3 cis-9, 12, 15), while the n6/n3 ratio decreased significantly in all tissues studied. Therefore, it can be concluded that supplementation in the diet of growing pigs is a potential way to increase the FA composition to the level required for healthier meat. Liu et al. [134] obtained similar results. They confirmed that the addition of 50 g/kg of expeller-pressed walnut kernel cake to the diet of fattening pigs promoted adipose deposition and improved pork quality during pig growth. In addition to the use of native oilseed species as a source of cheaper protein replacing soy protein in pig nutrition, the need to manage waste products, such as olive cake production, is also emphasized. Ferrer et al. [135] found in their study that one of the key factors in improving the sustainability of pig production is the use of agri-food industry by-products in feed, such as olive by-products. The agro-industrial olive sector produces large quantities of olive by-products, considered extremely toxic, with a highly adverse environmental impact. However, a holistic approach to the production process of co-products and the environmental impact throughout the entire production chain is crucial. To this end, an experiment was conducted to determine the effect of incorporating partially defatted olive cake into pig diets on growth, fecal microbiota, carcass quality, and gas emissions from slurry. The experimental diet included olive cake at a dose of 120 g/kg. There were no significant differences between the treatment groups in terms of yield, carcass quality, and microbial counts, with the exception of loin eye height muscle thickness, which was lower in the experimental animals compared with the control group (45.5 vs. 47.5 mm). The FA profile of subcutaneous fat did not differ between the treatment groups, but the concentration of MUFA was higher and PUFA was lower in the animals fed with the cake. Preliminary characterization of the pig slurry revealed differences only in the concentration of fiber, which was higher (*p* < 0.05) in the experimental group slurry. Regarding gas emissions, slurries from both treatments emitted similar amounts of NH_3_, CO_2_, CH_4_, and N_2_O, as well as biochemical methane potential values.

Liotta et al. [136] also further highlighted the growing interest in the incorporation of agro-industrial by-products into animal nutrition as a strategy to reduce both feed costs and the environmental burden associated with waste disposal. In their study, 72 Pietrain pigs were fed diets containing 0 (control), 50 g, or 100 g/kg olive cake as a partial substitute for wheat bran and soybean oil during the finishing period. The results demonstrated that the inclusion of olive cake did not impair growth performance and positively influenced several carcass and meat quality traits. In particular, pigs receiving olive cake exhibited reduced backfat thickness and lower intramuscular fat deposition compared with the control group (*p* < 0.05). Moreover, dietary supplementation altered the FA composition of both intramuscular and subcutaneous fat, leading to increased concentrations of MUFA and PUFA, as well as improvements in lipid quality indices, including the atherogenic index and thrombogenic index (*p* < 0.05). Importantly, the incorporation of olive cake did not negatively affect the organoleptic properties of pork, suggesting that this by-product can be successfully utilized as a functional feed ingredient.

The available literature indicates that OC can successfully serve as alternative protein and energy sources in pig nutrition, partially replacing conventional feed ingredients such as soybean meal without compromising production efficiency. Table 3 summarizes the effects of OC on pig health, performance, and carcass quality. In many cases, moderate inclusion levels of OC maintain or even improve growth performance, FCR, carcass characteristics, and meat quality. Furthermore, several oilseed by-products, including camelina, olive, walnut, hemp, linseed, and perilla cakes, have demonstrated the ability to beneficially modify the FA composition of pork by increasing the content of health-promoting n-3 PUFA and improving lipid quality indices.

## 6. Conclusions

In conclusion, based on the currently available evidence, camelina cake appears to have the strongest scientific support regarding its antioxidant and immunomodulatory properties in pig nutrition. Experimental studies consistently demonstrate its ability to modulate pro- and anti-inflammatory cytokine expression, enhance antioxidant enzyme activity, increase total antioxidant capacity, and regulate key signaling pathways involved in inflammation and lipid metabolism, including NF-κB and PPARγ. Comparatively strong evidence is also available for flaxseed cake, which has repeatedly been shown to improve antioxidant defense systems, attenuate inflammatory responses, and support intestinal health while simultaneously enhancing meat quality through modifications of the fatty acid profile.

For other oilseed by-products, including olive cake, grape seed cake, Gardenia seed cake, and fermented Jatropha curcas cake, the reported effects on oxidative status, immune function, gut microbiota, and production performance are promising but remain supported by a relatively limited number of studies, often conducted under specific experimental conditions. Consequently, their practical application requires further validation before firm nutritional recommendations can be established.

Across the available literature, the most consistent biological responses were generally observed at dietary inclusion levels of approximately 50–150 g/kg for flaxseed cake, 5–120 g/kg for camelina cake, and 50–100 g/kg for gardenia seed cake, whereas olive cake has shown beneficial effects primarily at 50–100 g/kg in finishing pigs. Nevertheless, the considerable variability in botanical origin, processing technology, residual oil content, concentrations of bioactive compounds, and anti-nutritional factors precludes the establishment of universal inclusion recommendations applicable to all OC.

Although the current body of evidence highlights the considerable potential of OC as sustainable functional feed ingredients capable of supporting redox homeostasis, immune competence, gut health, and meat quality while contributing to circular bioeconomy strategies, important knowledge gaps remain. Future research should prioritize standardized chemical characterization of OC, dose–response studies, and long-term validation under commercial production conditions. Particular attention should also be directed towards understanding the bioavailability and metabolism of bioactive compounds, evaluating interactions between oilseed-derived phytochemicals and the intestinal microbiome using multi-omics approaches (metagenomics, transcriptomics, metabolomics, and proteomics), and identifying biomarkers associated with oxidative balance and immune resilience. Furthermore, comparative studies evaluating different OC under standardized experimental conditions are essential for establishing evidence-based dietary recommendations and maximizing their contribution to sustainable and precise pig nutrition.

## Figures and Tables

**Table 2:** Effects of oilseed cakes on antioxidant status, and immune response in pigs.

Oilseed Cake	Animal Group	Study Duration	Inclusion Level (g/kg)	Immunology	Oxidative Stress	Blood Biochemistry	Additional Effects	References
Flaxseed cake	fatteners, (6 pigs/group)	42 days	0–100 g/kg	↓ TNF-α, IL-1β	↑ SOD, CAT, GPx	↓ cholesterol	100 g/kg flaxseed affected the antioxidant defense system	[115]
Camelina cake	Fatteners; 12 pigs/group	33 days	5–120 g/kg	↓ IL-1β, IL-6, TNF-α; ↑ IL-4	↑ TAC, SOD, CAT, GPx	Improved redox markers	Activation of PPARγ; inhibition of NF-κB	[98]
Grape seed cake/pomace	Fatteners; 6 pigs/group	24 days	50 g/kg	↓ IL-1β, TNF-α	↓ TBARS, MDA	Improved antioxidant profile	Rich source of polyphenols	[110]
Olive cake extract	Piglets (LPS challenge); 6 pigs/group	14 days	1/kg extract	↓ TNF-α, IL-6; ↑ IL-10	↑ SOD, GSH-Px; ↓ MDA, NO	↓ ALT, AST, LDH	Improved intestinal barrier integrity	[102]
Gardenia cake	Piglets; 60 pigs/group	47 days	50–100 g/kg	↓ IL-2, IL-8; ↑ IgA	↑ SOD, GSH-Px; ↓ MDA	Improved antioxidant profile	Dose-dependent immunomodulation	[103]
Fermented Jatropha cake	Growing pigs; 32 pigs/group	28 days	25–50/ g/kg	↓ IL-1, IL-6		Indirect reduction in oxidative stress	No data	[111]

Increase ↑ and decrease ↓.

**Table 3:** Effects of oilseed cakes on pig health and performance and carcass quality.

Oilseed Cake	Groups of Animals	Doses Content in Diet (g/kg)	Effect	References
Soybean	weaned pigs	200 g/kg soybean cake, fermented 80 g/kg soybean cake, toasted	↑ ADFI and ADG, ↓ diarrhea.	[116]
Camelina	fattening pigs	- Up to 50 g/kg - 150 g/kg - 180 g/kg - 50/100 /150 g	- ↔ on carcass characteristics; - ↓ ADFI, ADG, and BWG; - ↓ live weight; - ↓ hot carcass weight, dressing percentage, backfat thickness at the 10th rib, and ↑ the lean meat percentage. ↔ of feed on the percentage ↓ of carcass weight after chilling, meat pH at 45 min. and 24 h post-mortem, water absorption and marbling of pork chops, or belly firmness; - rich the pork with n-3 FA.	[119,120,130]
Rapeseed	Fattening pigs	20 g/kg	↔ BWG	[1]
Cottonseed	growing piglets	up to 300 g/kg	↓ FI and BWG, increased the weight of the heart, kidney, and liver.	[132]
Perilla			- ↑ the content of PUFA-FA and reduced the proportion of SA and USFA on meat; ADG ↑ supplementation ↔ carcass traits or meat quality, except for color brightness.	[133]
Pumpkin	fattening pigs	at 40 g/kg and70 g/kg	↔ on health and production performance	[132]
Olive	fattening pigs	- 120 g/kg - 50 g/kg or 100 g/kg/	- ↔ on terms of yield, carcass quality, and microbial counts, with the exception of muscle thickness; - ↔ of FA on meat; 50 g ↑ BW and ↓ FCR. 50 g and 100 g/kg impact ↓ the thickness of backfat and intramuscular fat and modified FA composition ↑ MUFA and PUFA and ↑ quality indicators; - higher content of n-3 FA, resulting in a lower ratio of n-6 to n-3 PUFAs; - naturally improve the nutritional value of the meat and ↑ the value of the by-product.	[135,136]
Walnut	Fattening pigs	50 g kg	promoted adipose deposition and improved pork quality during pig growth	[134]
Flaxseed fermented	post-weaned piglets (35 days old)	80 g/kg	↑ BWG, ADG, FCR, and diarrhea score, ↔ ADFI, compared with 80 g unfermented flaxseed cakes	[116]
Hemp and linseed	fattening pigs	mixture of domestic protein, i.e., peas, field beans, sunflower meal, and hemp and linseed cake,	- ↓ ADG, higher FI, and thus a less favorable FCR - ↑ backfat thickness and lower loin eye height and carcass meatiness - ↑ weight of the most commercially valuable primal cuts	[128]
Amarula	fattening pig	50,100,150 and; 200 g/kg; - increasing levels of Amarula cake	- ↔ ADG - ↓ FI and without negatively affecting growth, nutrient digestibility, and carcass characteristics. - ↓ linear relationship was found between ↑ levels of Amarula cake, ADG and FCR, carcass length, hot and cold carcass weight. stomach weight, backfat thickness, juice leakage and hepato-somatic index increased linearly	[126,131]

Increase ↑ and decrease ↓.

## Data Availability

No new data were created or analyzed in this study. Data sharing is not applicable to this article.

## Abbreviations

The following abbreviations are used in this manuscript:

ADFI	Daily feed intake
ADG	Average daily gain
ALA	alpha-linolenic acid
AST	aspartate aminotransferase
BW	Body weight
BWG	Body weight gain
CAT	Catalase
DBWG	Daily body weight gain
DM	Dry matter
DPPH	2,2-diphenyl-1-picrylhydrazyl
FA	Fatty acids
FCR	Feed conversion ratio
FI	Feed intake
FRAP	Ferric reducing antioxidant power/ability
GLA	γ-linolenic acid
GPx	glutathione peroxidase
Ig	Immunoglobulin
IL	Interleukin
MDA	Malondialdehyde
MUFA	Monounsaturated fatty acids
NO	nitric oxide
PUFA	Polyunsaturated fatty acids
OC	Oilseed cake
ROM	Reactive oxygen metabolites
ROS	Reactive oxygen species
SFA	Saturated fatty acids
SOD	Superoxide dismutase
TBA	Thiobarbituric acid
TBARS	Thiobarbituric acid reactive substances
TFC	total flavonoid content
TPC	Total phenolic content
TNF-α	Tumor necrosis factor
UFA	Unsaturated fatty acids
VFA	Volatile fatty acid
WHC	Water-holding capacity
